# Fc receptor engagement of HIV-1 Env-specific antibodies in mothers and infants predicts reduced vertical transmission

**DOI:** 10.3389/fimmu.2022.1051501

**Published:** 2022-12-12

**Authors:** Brittani M. Barrows, Shelly J. Krebs, Ningbo Jian, Michelle Zemil, Bonnie M. Slike, Vincent Dussupt, Ursula Tran, Letzibeth Mendez-Rivera, David Chang, Anne Marie O’Sullivan, Brendan Mann, Eric Sanders-Buell, Zhanna Shubin, Matt Creegan, Dominic Paquin-Proulx, Philip Ehrenberg, Agnes Laurence-Chenine, Kriengkrai Srithanaviboonchai, Rasmi Thomas, Michael A. Eller, Guido Ferrari, Merlin Robb, Venigalla Rao, Sodsai Tovanabutra, Victoria R. Polonis, Lindsay Wieczorek

**Affiliations:** ^1^ U.S. Military HIV Research Program, Walter Reed Army Institute of Research, Silver Spring, MD, United States; ^2^ Henry M. Jackson Foundation for the Advancement of Military Medicine, Inc., Bethesda, MD, United States; ^3^ Department of Biology, The Catholic University of America, Washington, DC, United States; ^4^ Department of Community Medicine, Chiang Mai University, Chiang Mai, Thailand; ^5^ Department of Surgery, Duke University School of Medicine, Durham, NC, United States

**Keywords:** vertical transmission, antibody dependent cellular cytotoxicity, neonatal Fc receptor, passive immunity, CRF01_AE HIV, Thailand, protective immunity, pregnancy

## Abstract

**Introduction:**

Infants acquire maternal antibodies by Fc receptor transcytosis across the placenta during pregnancy. Fc receptors are expressed on immune cells and are important for activation of effector cell functions.

**Methods:**

In this study, we evaluated Fc receptor engagement and ADCC activity of plasma binding antibodies from human immunodeficiency virus-1 (HIV) -infected mothers and to identify factors that may contribute to protection from HIV vertical transmission.

**Results:**

HIV-specific binding and Fc receptor engagement of plasma antibodies varied between mothers by transmission status and infants by infection status. Non-transmitting (NT) mothers and HIV-uninfected infants had antibodies with higher neonatal Fc receptor (FcRn) and FcγR engagement, as compared to transmitting (T) mothers and HIV+ infants, respectively. A significant inverse correlation between plasma antibody FcRn and FcγR engagement was observed for T mothers, but not NT mothers. Conversely, a significant direct correlation was observed between plasma antibody FcRn and FcγR engagement for HIV- infants, but not for HIV+ infants. Consequently, we observed significantly higher plasma antibody ADCC potency and breadth in HIV- infants, as compared to HIV+ infants. However, no differences in overall ADCC potency and breadth were observed between mothers. FcRn-engagement of HIV-specific antibodies in both mothers and infants predicted a lack of vertical transmission of HIV.

**Discussion:**

This study indicates that HIV-uninfected infants acquire HIV-specific antibodies with greater Fc receptor engagement and thus, greater ADCC capacity.

## Introduction

Mother-to-child transmission (MTCT) of HIV remains a global health concern due to high incidence and low treatment coverage for adolescent women (1, 2). Pregnant women infected with HIV-1 transmit the virus to their infants during pregnancy, labor and delivery, or through breastfeeding, and higher maternal viral loads associate with higher transmission rates (3). Without antiretroviral therapy (ART) during pregnancy, transmission occurs at a frequency of 25%, implying a protective mechanism may be active during pregnancy and birth (4). Administration of ART during pregnancy can reduce transmission rates to <1%. However, ART is not always available, may be less effective against increasing drug resistant HIV strains, and can have harmful health consequences for infants (4, 5). A deeper understanding of potential natural protection from MTCT may inform improved prevention and treatment options for HIV infected pregnant women and their at-risk children, and potentially contribute to other preventative technologies, including vaccine strategies.

During pregnancy, maternal antibodies are transported across the placenta and provide infant protection against pediatric infectious diseases (6, 7). Maternal IgG transverses three placental barriers before entering fetal circulation, including a layer of syncytiotrophoblast cells, then placental fibroblasts and Hofbauer cells, and finally a layer of fetal endothelial cells (8). The neonatal Fc receptor (FcRn) is critical for transplacental transport of IgG and binds to maternal IgG Fc in the endosome of the syncytiotrophoblasts (9). Expression of noncanonical Fc receptors (FcR) have been found on all three placental cell layers (10) and could assist in further IgG transport (5). This process of antibody transport by IgG Fc-FcR binding may bias IgG selection and therefore infant antibody Fc-mediated antibody functions.

Non-neutralizing antibody effector functions are mediated by FcR expressed on immune cells. FcγR2a, FcγR2b, FcγR3a, and FcγR3b are four low-affinity FcRs for IgG containing immune complexes (11). Activating FcγR2a (H131, high affinity; R131, low affinity) and inhibitory FcγR2b are expressed on neutrophils, eosinophils and monocytes and are involved in phagocytosis of IgG2 immune complexes. FcγR3a (V158, high affinity; F158, low affinity) is expressed on natural killer (NK) cells and mediates antibody dependent cellular cytotoxicity (ADCC) after engaging antibody-bound infected target cells. FcγR3b is expressed on neutrophils and involved in cellular recruitment at sites of inflammation. Plasma ADCC activity was a correlate of vaccine protection in the RV144 vaccine trial performed in Thailand (12), yet it is unclear whether ADCC or other Fc-mediated, non-neutralizing antibody activities are protective against vertical transmission of HIV. Further investigation is warranted to better understand the effector functionality of placentally transferred HIV antibodies.

In this study, we evaluated the HIV Env-specificity, Fc receptor binding and Fc-mediated antibody functions of mother and infant plasma antibodies to better understand the role of humoral immunity in MTCT. For this study, we utilized plasma samples from a Thai cohort for which we previously observed significantly higher neutralizing antibody titers in HIV-uninfected (HIV-) infants, as compared to HIV+ infants, and interestingly, for mothers that transmitted HIV to their infants (T), as compared to mothers that did not transmit HIV to their infants (NT) (13). This discrepancy indicated a potential bias in antibody transplacental selection that we further explore in this study. Here, we evaluated the non-neutralizing antibody effector functions of ADCC and ADCP in plasma collected from HIV+ pregnant women at delivery and their infants at 2 months after birth. Additionally, we characterized plasma antibody antigen-specificity and FcR-reactivity for comparison with functional plasma activity and transmission or infection status.

We observed significantly higher ADCC potency and breadth in HIV- infants, as compared to HIV+ infants, however no differences were observed between mothers. Overall, NT mothers and HIV- infants had higher HIV-specific binding antibodies and FcR-engagement than T mothers and HIV+ infants, respectively. A significant inverse correlation between plasma antibody FcRn and FcγR engagement was observed for T mothers, but not NT mothers. Conversely, a significant direct correlation was observed between plasma antibody FcRn and FcR engagement for HIV- infants, but not for HIV+ infants. FcRn-engagement of HIV-specific antibodies in both mothers and infants predicted a lack of vertical transmission of HIV. These results support the role of FcRn in mediating the transfer of antibodies mediating Fc effector functions that contributed to protection against MTCT.

## Results

Mother and infant plasma samples were evaluated from an HIV perinatal transmission study conducted at the Lampang Hospital in Lampang, Thailand between 1996 and 1998 (13). Mother plasma samples included in this analysis were from HIV+ pregnant women with a confirmed subtype CRF01_AE HIV-1 infection. All mothers were 30 years of age or less and were ART-naïve during pregnancy and at delivery. Plasma analyzed in this study were collected from the mothers at time of delivery, and from their infants two months after birth. Both mothers and infants were asymptomatic at the time of sample collections (14). Higher maternal viral load (VL) was previously determined to be predictive of increased odds of transmission (odds ratio [OR] = 8.3, 95% confidence intervals [CI] = 2.9, 24.1) (13). To minimize the impact of VL on our analysis, NT mothers were selected to have similar viral loads and CD4 T-cell counts as the T mothers, however due to sample restrictions, exact matching was not possible.

### Mother and infant plasma antibody Fc mediated activity

**Figure 1:**
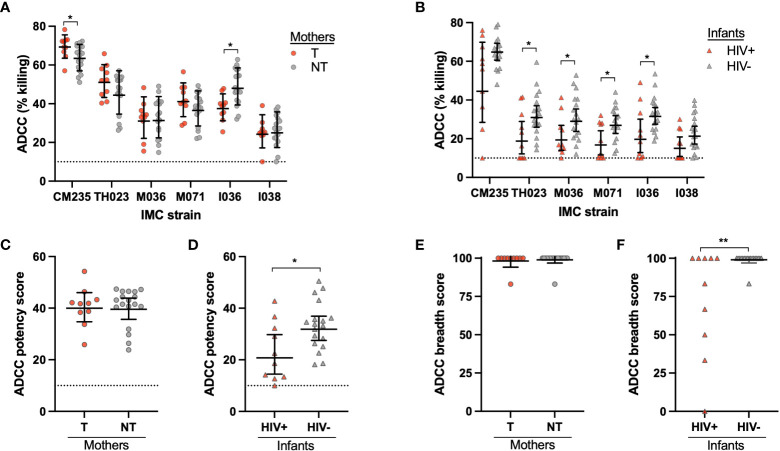
Mother and infant plasma ADCC activity. ADCC activity was measured for the **(A)** transmitting (T) and non-transmitting (NT) mothers and **(B)** HIV+ and uninfected (HIV-) infants using six CRF01_AE HIV strains in the ADCC-Luciferase assay. CRF01_AE HIV IMC strains CM235, TH023, M036, M071, I036, and I038 were used; M036 and M071 were originally isolated from the maternal cohort, and I036 and I038 were originally isolated from the infant cohort. The ADCC potency was calculated as the geometric mean of ADCC % killing for all IMC strains for the **(C)** mothers and **(D)** infants. The ADCC breadth score was determined as the percentage of IMC for which ADCC activity was observed for the **(E)** mothers and **(F)** infants. The dotted line at 10% killing indicates the negative cut off value for the assay. Significant differences were determined by Mann-Whitney test and are indicated by * above the plots; * = p < 0.05, ** = p < 0.005.

ADCC activity was evaluated for 10 T mothers and 18 NT mothers, as well as their infants, including 10 HIV+ and 18 HIV- infants. Mother and infant plasma ADCC activity was tested using HIV+ target cells with PBMC effector cells from a selected healthy donor. Target cells were separately infected with 6 different CRF01_AE HIV infectious molecular clones (IMCs), including two widely used CRF01_AE strains (CM235 and TH023), two strains that were isolated from HIV+ mothers in the cohort (M036 and M071) and two strains that were isolated from HIV+ infants in the cohort (I036 and I038). Overall, few differences were found in ADCC activity between NT and T mothers, with variations in ADCC activity for both CM235 and I036 (**Figure 1A**). While the NT mothers had significantly higher ADCC activity against the infant I036 IMC (p=0.005), no difference was observed between NT and T mothers against the matched maternal IMC strain, M036. Conversely, the HIV- infants demonstrated significantly higher ADCC activity against 4 out of the 6 IMC strains, including TH023 (p=0.030), M036 (p=0.039), M071 (p=0.017) and I036 (p=0.041) (**Figure 1B**). We further created an ADCC potency score by calculating the geometric mean of % killing for all 6 IMCs, as well as an ADCC breadth score by calculating the percentage of IMC for which ADCC activity was observed. HIV- infants had a significantly higher ADCC potency score (p=0.032, **Figure 1D**) and ADCC breadth score (p=0.005, **Figure 1F**) as compared to HIV+ infants, however, no difference was observed in ADCC potency or breadth between T and NT mothers (**Figures 1C, E**, respectively).

Plasma antibody dependent cellular phagocytosis (ADCP) activity was also evaluated for comparison between Fc-mediated antibody functions. ADCP is mediated by monocytes or macrophages through binding of the FcγR1 or FcγR2a receptor to the Fc region of IgG-bound targets. Maternal and infant samples were evaluated against M036 (cohort T mother) gp140 protein for ADCP activity and separated by transmission status. No significant differences were observed between NT and T mother or HIV- and HIV+ infant ADCP activity (**Figure S1**).

### Mother and infant plasma antibody IgG, Env specificity and Fc reactivity

**Figure 2:**
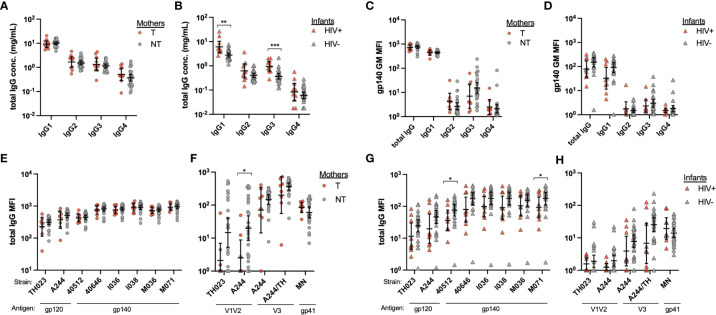
Mother and infant plasma total IgG and HIV antigen-specific IgG. Total IgG1-4 were measured from plasma from **(A)** transmitting (T) and non-transmitting (NT) mothers and **(B)** HIV+ and uninfected (HIV-) infants by IgG subclass ELISA. HIV antigen specific IgG was evaluated by Luminex multiplex assays using multiple HIV Env antigens and anti-IgG subclass as secondary antibodies. The geometric mean of the mean florescence intensity (MFI) for six gp140 proteins was determined for **(C)** mother and **(D)** infant total IgG and IgG subclasses 1-4. Total IgG binding of plasma from mothers **(E**, **F)** and infants (G and H) to specific HIV antigens is shown for Env **(E**, **G)** and Env domains **(F**, **H)**. Significant differences were determined by Mann-Whitney test and are indicated by * above the plots; * = p < 0.05, ** = p < 0.005, *** = p < 0.0005.

To determine whether the increased ADCC activity in HIV- infants was a result of increased transfer of total IgG, we next examined total IgG and IgG subclass concentrations by ELISA. An additional 8 NT mother and HIV- infant samples were included. T and NT mothers had similar total IgG1-4 concentrations (**Figure 2A**), while HIV+ infants had significantly higher IgG1 (p=0.002) and IgG3 (p=0.0005) concentrations as compared to HIV- infants (**Figure 2B**). We next evaluated HIV Env reactivity of total plasma IgG and IgG1-4 using a panel of 6 CRF01_AE gp140 proteins and calculated the geometric mean of the plasma antibody binding magnitude using a Luminex multiplex assay. No significant differences in overall antibody binding magnitude of total IgG or IgG1-4 to HIV Envs were observed between NT and T mothers (**Figure 2C**) or HIV- and HIV+ infants (**Figure 2D**). We evaluated total IgG reactivity to HIV Env gp120 and gp40 proteins, and Env domain antigens, including V1V2, V3 and gp41. No significant differences were found in IgG binding to HIV Env or Env domains between T and NT mothers, except for the A244 strain V1V2 protein for which NT mothers had significantly higher binding antibody responses (p=0.008) (**Figures 2E, F**). Similarly, overall there were no significant differences found in IgG binding to HIV Env or Env domains between HIV- and HIV+ infants, with few exceptions (**Figures 2G, H**). HIV- infants demonstrated significantly higher binding antibody responses to the 40512 and M071 gp140 proteins, as compared to HIV+ infants (p=0.022 and p=0.050, respectively, **Figure 2G**).

**Figure 3:**
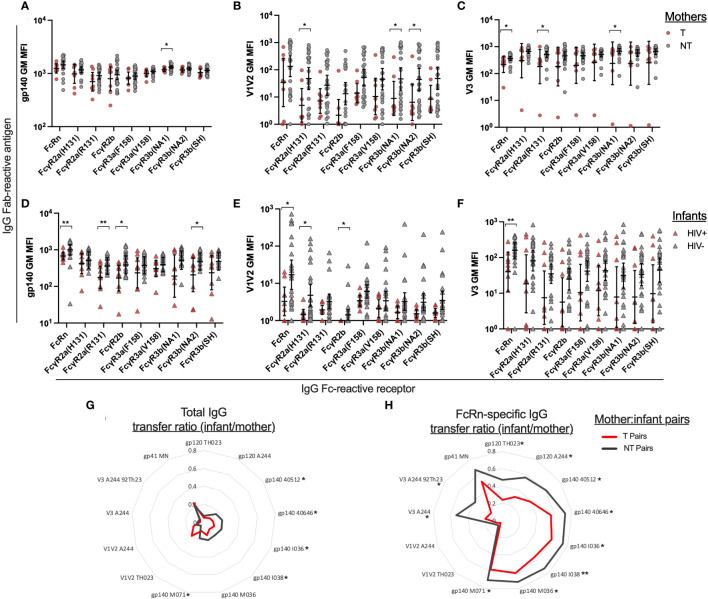
Plasma IgG Fc receptor binding and transfer efficiency of Env-specific plasma IgG from mothers to infants. Using Luminex multiplex assays, plasma antibodies were evaluated for HIV Env antigen binding and Fc receptor engagement. The geometric mean of the mean florescence intensity (MFI) was determined for six gp140 proteins, two V1V2 proteins, and two V3 antigens. Fc Receptor (FcR) engagement is shown for transmitting (T) and non-transmitting (NT) mother plasma antibodies binding to HIV antigens **(A)** gp140 **(B)** V1V2 and **(C)** V3. Fc Receptor (FcR) engagement is shown for HIV+ and uninfected (HIV-) infant plasma antibodies binding to HIV antigens **(D)** gp140 **(E)** V1V2 and **(F)** V3. Binding antibody transfer ratios were calculated by dividing the infant MFI for a specific antigen/FcR combination by the matched mother MFI for the same antigen/FcR combination. The geometric mean of the transfer ratio is shown in radar plots for the transmitting mother-infant pairs (red line) and non-transmitting mother-infant pairs (gray line) for individual antigens for **(G)** total IgG and **(H)** FcRn-specific IgG. Significant differences were determined by Mann-Whitney test and are indicated by * above the plots; * = p < 0.05, ** = p < 0.005.

Next, we evaluated FcR engagement of gp140, V1V2 and V3 specific IgG in T and NT mother and their HIV+ and HIV- infants, respectively. We produced and utilized the neonatal receptor FcRn, as well as receptors known to function for varying Fc-mediated functions, including FcγR2a, FcγR2b, FcγR3a, and FcγR3b. Overall, there were no significant differences between NT and T mothers in gp140-specific binding reactivity for FcRs, with significant difference only observed for FcγR3b(NA1) (p=0.040; **Figure 3A**). Interestingly, NT mothers demonstrated higher FcR engagement of antibodies targeting V1V2 and V3 compared to T mothers, however a wide range of binding antibody responses was observed for both groups (**Figures 3B, C**). HIV- infants demonstrated overall higher engagement of FcR to antibodies targeting gp140, V1V2 and V3 compared to HIV+ infants. Mostly notably, HIV- infants demonstrated significantly higher engagement of FcRn to antibodies targeting gp140 (p=0.004; **Figure 3D**), V1V2 (p=0.046; **Figure 3E**), and V3 (p=0.003; **Figure 3F**) antigens.

We then compared the paired mother and infant binding antibody responses observed for gp140, V1V2 and V3-spedific total IgG and FcRn-specific IgG (**Figure S2**). To summarize these data, a mother to infant transfer score was calculated by dividing the infant’s binding antibody response value by their mother’s binding antibody response value for each antigen-detector combination. We calculated the geometric mean of the transfer scores for the T/HIV+ mother/infant pairs and NT/HIV- mother/infant pairs and show the results in radar plots for total IgG and FcRn-specific IgG (**Figures 3G, H**). Overall, NT/HIV- pairs had higher transfer ratios than T/HIV+ pairs, except for A244 strain V1V2-specific IgG, for which minimal binding was observed for T mothers. NT/HIV- pairs have significantly higher transfer ratios than T/HIV+ pairs for gp120, gp140 and V3-specific total IgG and FcRn-specific IgG, as indicated by the * next to the antigen labels in **Figures 3G, H**.

### Relationships between plasma antibody FcR reactivities and ADCC activity

**Figure 4:**
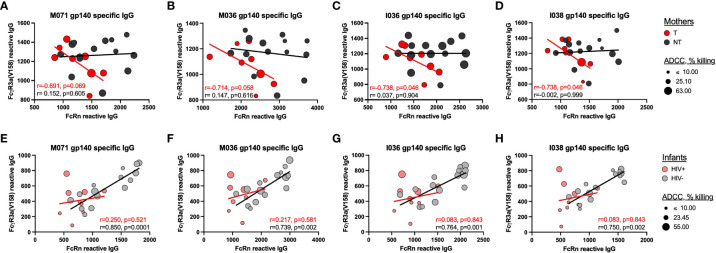
Relationship between homologous-Env specific FcRn and FcyR3a(V158) reactive IgG and plasma ADCC. The correlations between gp140-specific FcRn-reactive plasma IgG and gp140-specific FcyR3a(V158)-reactive plasma IgG were evaluated for **(A–D)** mothers and **(E–H)** infants using homologous gp140 cohort proteins, including **(A**, **E**) M071 gp140, B and **(F)** M036 gp140, **C**, **(G)** I036 gp140 and **D**, **(H)** I038 gp140. The trend lines and inset spearman correlation coefficients are shown to demonstrate the relationship between homologous gp140-specific FcRn-reactive plasma IgG and FcyR3a(V158)-reactive plasma IgG for the transmitting mothers (T, red), non-transmitting mothers (NT, dark gray), HIV+ infants (pink) and uninfected infants (HIV-, light gray). The size of the symbol reflects the magnitude of ADCC activity against the homologous Env IMC strain.

We then evaluated the relationship between plasma antibody binding responses and ADCC activity against homologous cohort Env gp140 antigens and IMC, respectively. Utilizing four gp140 proteins produced from two mother and two infant Env strains, we evaluated the correlations between gp140-specific IgG with FcRn reactivity and gp140-specific IgG with FcγR3a(V158) reactivity (**Figure 4**). FcγR3a was chosen due to its known role in mediating ADCC. We observed significant negative correlations between gp140-specific IgG with FcRn reactivity and gp140-specific IgG with FcγR3a(V158) reactivity for T mothers, but not NT mothers, as indicated by the trend lines and spearman correlation coefficients shown in the chart insets (**Figures 4A-D**). Interestingly, we observed significant positive correlations between gp140-specific IgG with FcRn reactivity and gp140-specific IgG with FcγR3a(V158) reactivity for HIV- infants, but not HIV+ infants (**Figures 4E-H**). The size of each symbol reflects the magnitude of the plasma ADCC activity mediated against the homologous Env IMC. Infants that did not mediate ADCC activity (small symbols), consistently have low gp140-specific FcRn reactive IgG and gp140-specific FcγR3a(V158) reactive IgG.

Due to the consistency of trends across gp140 antigens, we created a cohort gp140 binding score to further evaluate the correlation between gp140 FcRn-reactivity and IgG Fc reactivity with other FcRs (**Table 1**). For this analysis we used a larger number of samples, as indicated in the table, since we did not incorporate ADCC activity into the analysis. However, for NT mothers we we observed negative correlations between FcRn-reactivity and FcR-reactivity for FcγR2a(H131), FcγR3a(F158), FcγR3a(V158) and FcγR3b(NA2). While for NT mothers, we observed significant positive correlations between FcRn-reactivity and FcR-reactivity for FcγR2b and FcγR3b(NA1). No significant correlations were observed for HIV+ infants, while significant direct correlations between FcRn and FcR-reactive IgG were observed for HIV- infants for all FcRs, except FcγR3a(F158). This further supports the role of FcRn binding in selection of IgG with increased FcR poly-reactivity.

**Table 1:** Correlation between gp140-specific FcRn-reactive plasma IgG and gp140-specific FcR-reactive plasma IgG.

		FcγR2a(H131)	FcγR2a(R131)	FcγR2b	FcγR3a(F158)	FcγR3a(V158)	FcγR3b(NA1)	FcγR3b(NA2)	FcγR3b(SH)
												**Spearman correlation**	
T mothers (N=8)	FcRn	-0.62	0.05	0.21	-0.52	-0.71	0.71	-0.60	-0.07			coefficient	p value
NT mothers (N=25)	FcRn	0.26	0.15	**0.76**	0.19	0.06	**0.48**	0.20	0.23			1	>0.05
HIV+ infants (N=9)	FcRn	0.25	0.12	0.12	0.12	0.15	0.22	0.25	0.12			0	**<0.05**
HIV- infants (N=25)	FcRn	**0.44**	**0.50**	**0.73**	0.32	**0.73**	**0.48**	**0.47**	**0.60**			-1	
											

The bold numbers indicate spearman correlation p values <0.05.

The color shading reflects the value of the spearman correlation coefficient. A value of 1 is colored as green, a value of 0 is white, and a value of -1 is colored as orange.

Infant PBMC were then assessed for common FcγR3a single nucleotide polymorphism (SNP) at amino acid position 158 (F or V) which are known to influence IgG binding affinity and ADCC functionality, and for FcγR3a expression levels for comparison with infection status. FcγR3a SNP differences were not observed between HIV+ (n=9) and HIV- (n=15) infants, although a limited number of samples was evaluated, based on availability (**Figure S3A**). Immunophenotyping of infant PBMC was conducted to verify that the infants have NK cell populations with mature markers indicative of cytotoxicity, as previously reported (15, 16). While we observed cytotoxic NK cell populations in both HIV- and HIV+ infants, no significant differences were observed in the frequency of CD56^dim^ NK cells or in the NK cell populations with high cytotoxic potential (CD56^dim^+, CD57+; **Figure S3B, C** respectively).

### Multivariate statistical analysis of antibody features associated with reduced vertical transmission

**Figure 5:**
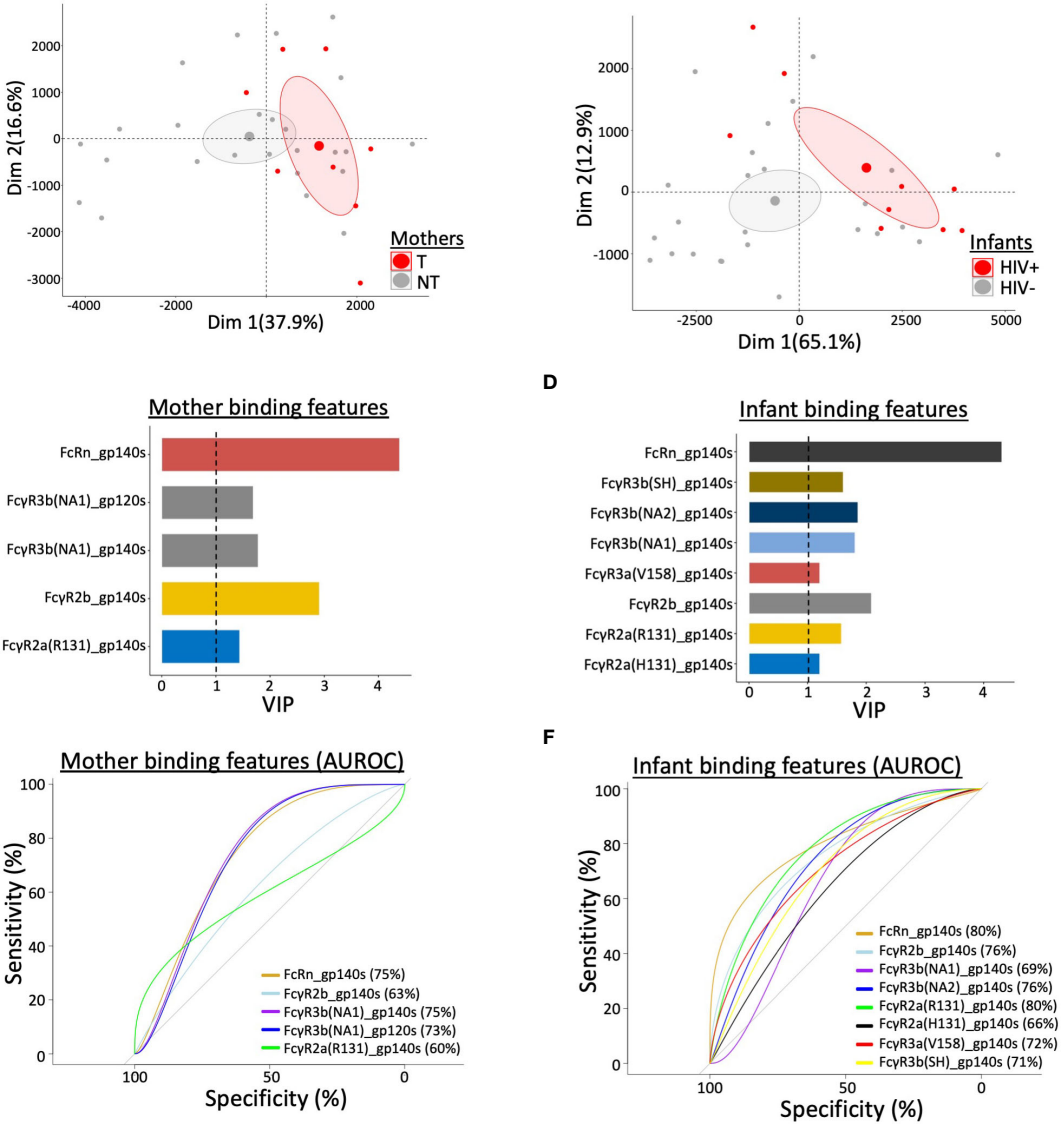
Multivariate statistical analysis of antibody features that predict vertical transmission of HIV. Principal component analysis (PCA) of antibody profiling stratified by **(A)** mother’s transmission status for transmitting (T) and non-transmitting (NT) mothers and **(B)** infant’s HIV status for HIV+ and uninfected (HIV-) infants. Ellipses correspond to 95% confidence intervals for each group. Variable Important Projection (VIP) scores, through Partial Least Square Discriminant Analysis (PLSDA), indicate the capability of contributing to the model in terms of separating **(C)** mothers by transmission status and **(D)** infants by infection status. VIP score > 1 was used as threshold to select the most contributing features among all binding features in study. Area under the receiver operating characteristic curve (AUROC) shows the predictive ability of selected features to separate **(E)** T and NT mothers, and **(F)** HIV+ and HIV- infants. AUC values are indicated in each panel.

Finally, to gain an understanding of which antibody binding features were predictive of reduced transmission, we performed multivariate partial least squares-discriminate analysis (PLS-DA) to develop a data model (17, 18). Separation was observed across the antibody features between T and NT mothers (**Figure 5A**) and between HIV+ and HIV- infants (**Figure 5B**), marked by elevated engagement of FcRn in the NT mothers and HIV- infants. Specifically, NT mothers and HIV- infants exhibited immune profiles enriched in higher levels of FcR engagement across HIV Env proteins, with FcRn engagement of both mother (**Figure 5C**) and infant (**Figure 5D**) antibody as the most predictive feature of lower transmission. A variable importance in projection (VIP) score of >1 was utilized for the cutoff of predictive feature selection (**Figures 5C, D**). In addition, receiver operating characteristic (ROC) plots showed that FcRn and FcγR3b were effective at distinguishing NT and T mothers, achieving an area under the curve (AUC) of 0.75 (**Figure 5E**). FcRn and FcγR2b engagement of HIV Env binding antibodies were also able to discriminate between HIV- and HIV+ infants, with an AUC of >0.80 for FcRn alone (**Figure 5F**). Strikingly, high FcRn engagement of binding antibodies that recognized multiple gp120 and gp140 antigens was the most predictive factor in reduced vertical transmission of HIV-1 in both mothers and infants.

## Discussion

Transplacental antibody transport is critical for protection of infants against pathogens during the first few months of life. The functional activity of the maternal antibodies, as well as their ability to traverse the placenta, will impact the quality of the infant’s immune response. During HIV+ pregnancies, virus can pass through the placenta, along with antibodies, and infect the fetus. Perinatal transmission occurs at a rate of 25% without intervention during pregnancy or delivery (19, 20), indicating the existence of a naturally occurring protection mechanism. In this study, we evaluated HIV+ mothers and their HIV+ or HIV- infants for plasma antibody ADCC activity, binding antibody specificity and FcR engagement to elucidate the role they may play in protection against MTCT of HIV.

Plasma antibody ADCC activity has been shown to be protective against HIV infection or pathogenesis in many settings (21). ADCC of HIV-infected cells has correlated with protection in vaccine studies (22, 23) and improved outcomes in natural infection (24, 25). A previous study evaluating infant HIV transmission through breast milk identified that HIV-exposed uninfected infants had higher ADCC and neutralizing antibodies, against their mother’s HIV strain, as compared to HIV-exposed infected infants (26). Additionally, the HIV+ infants with high ADCC activity had lower morbidity up to 1 year after birth than HIV+ infants with low ADCC activity (26). Separate studies have shown that infant plasma ADCC activity, but not ADCC activity of the mother’s plasma, is associated with improved HIV+ infant survival (27, 28). In this study, we showed that HIV- infants had significantly higher overall ADCC magnitude and breadth, as compared to HIV+ infants, while no difference was observed between T and NT mothers. One interesting exception to this was that significantly higher plasma ADCC activity was observed for NT mothers, as compared with T mothers, against the infant IMC strain, I036, but not the corresponding mother IMC strain, M036. This indicates improved reactivity of the NT mother plasma against this perinatally transmitted infant HIV strain, as compared to the corresponding mother HIV strain.

We then explored the plasma IgG Env-specificity that may be associated with transmission or infection status. Firstly, we qualified total plasma IgG by subclass and identified significantly higher total IgG1 and IgG3 in the HIV+ infant plasma, as compared to HIV- infants. HIV-exposed children have been shown to have abnormal IgG subclass distribution (29, 30). In a prior small comparison, it was shown that HIV+ infants had hypergammaglobulinemia and higher total IgG1 and IgG3 as compared to HIV-exposed uninfected children (29). Our results similarly demonstrate an imbalance between HIV-exposed infected and HIV-exposed uninfected infants in IgG transport or early IgG production for IgG1 and IgG3; it is unclear whether this contributed to or was caused by the HIV infection. Interestingly, we observed almost no difference in levels of Env-specific total IgG or IgG1-4 for the mothers or infants. However, NT mother had significantly higher V1V2-specific binding antibodies than T mothers. The partial protection observed in the RV144 vaccine trial in Thailand was correlated with V1V2-specific IgG shown to mediate ADCC (12, 31). However, V1V2 and V3-specific IgG were not predictive features of non-transmission in this study.

The Fc reactivity of gp140, V1V2 and V3-specific IgG was also evaluated. Overall, we observed higher FcR-reactivity for NT mothers and HIV- infants, as compared to T mothers and HIV+ infants, respectively. We consistently observed significantly higher FcRn reactivity for HIV- infants gp140, V1V2 and V3-specific IgG and NT mothers V3-specific IgG as compared to HIV+ infants and T mothers, respectively. Due to the increased FcRn reactivity observed for the NT mother and HIV- infant plasma, we calculated an antigen-specific IgG transfer ratio from matched mother to infant to evaluate the relationship between total IgG and FcRn-reactive IgG between T/HIV+ and NT/HIV- mother-infant pairs. We observed higher transfer ratios for NT/HIV- pairs, as compared T/HIV+ pairs, with significantly higher ratios for gp140-specific total IgG (5 out of 13 antigens) and gp120, gp140 and V3-specific FcRn-reactive IgG (10 out of 13 antigens). Maternal HIV infection, independent of maternal hypergammaglobulinemia, has previously been shown to decrease antibody transfer to infants (32, 33). However, differences between T and NT mothers have not been previously demonstrated.

**Figure 6:**
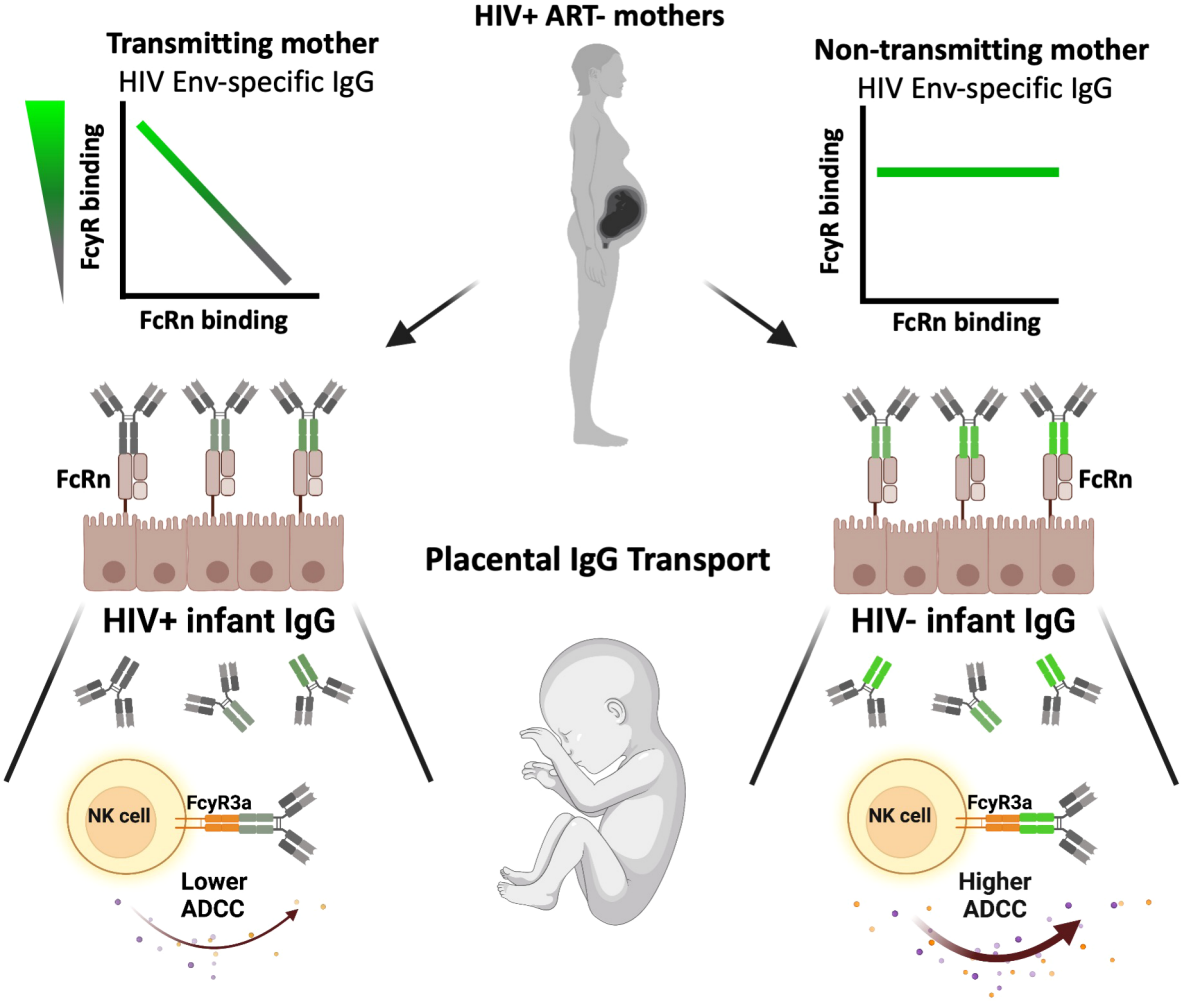
Graphical summary of the impact of maternal antibody profile on transplacental IgG transport, infant immunity and vertical transmission. On the left, transmitting mothers demonstrated a significant inverse correlation between FcRn-reactive and FcγR-reactive Env-specific plasma IgG (top), resulting in FcRn-mediated transplacental transport of IgG with lower FcγR-reactivity (middle), and therefore lower infant plasma IgG ADCC activity (bottom). Conversely, on the right, non-transmitting mothers demonstrated no significant relationship between FcRn-reactive and FcγR-reactive Env-specific plasma IgG (top), resulting in FcRn-mediated transplacental transport of IgG with unbiased FcγR-reactivity (middle), and therefore infant with higher plasma IgG ADCC activity as compared to the HIV+ infant counterparts (bottom).

We further evaluated the relationship between FcRn selectivity and ADCC activity by comparing plasma IgG FcRn-reactivity, FcγR3a(V158)-reactivity and ADCC activity against cohort Env antigens. For both HIV- and HIV+ infants, we observed a shift towards positive correlative relationships between gp140-specific FcRn-reactive IgG and gp140-specific FcγR3a(V158)-reactive IgG. For HIV- infants, a significant positive association was observed for all cohort Env strains, while no association was observed for HIV+ infants. Further, low levels of gp140-specific FcRn-reactive and FcγR3a(V158)-reactive IgG were observed for all infants that did not mediate ADCC activity against the homologous Env IMC. Interestingly, we observed a significant negative correlation between gp140-specific FcRn-reactive IgG and gp140-specific FcγR3a(V158)-reactive IgG for T mothers but not NT mothers, for all 4 cohort Env strains. T mothers with high levels of FcRn-reactive IgG had low levels of FcγR3a(V158)-reactive IgG, indicating a greater potential for transplacental transport, but a lower capacity of IgG to mediate ADCC. Conversely, T mothers with low levels of FcRn-reactive IgG had high levels of FcγR3a(V158)-reactive IgG, indicating a lower potential for transplacental transport of IgG capable of mediating ADCC. This observation highlights a quality of the T mother’s IgG that may contribute to lack of infant protection, as demonstrated graphically in **Figure 6**.

This sieving of Fc-specific IgG across the placenta has been previously demonstrated (5, 34). Infants have been shown to have a higher proportion of IgG with di-galactosylated Fc-glycans and higher binding to FcRn and FcγR3a receptors than their mothers (34). We did not evaluate antibody glycosylation patterns in this study due to sample volume limitation. However, our results are consistent with this observation in that greater IgG FcRn binding leads to greater antibody transfer efficiencies and results in infant IgG with enhanced ADCC activity and neutralizing activity, as previously shown (13). Our study further demonstrates the role of FcRn-selectivity of IgG, and the poly FcR-reactivity of mother antigen-specific IgG, in protection of the fetus during HIV+ pregnancy. HIV Env-specific IgG with FcRn-reactivity was predictive of non-transmission for both mother and infants in this study. Additional predictive features of non-transmission included mother and infant Env-specific IgG reactive for multiple FcR, indicating the importance of transplacental transport of poly-reactive IgG in infant protection.

It is important to note that samples included in this analysis were from a study conducted prior to standardization of ART for prevention of MTCT of HIV. All maternal samples were drug-naive. Thailand has since utilized ART to become the first country to achieve WHO standards for elimination of MTCT (35). However, several countries still struggle with initial or continued access to ART. HIV vaccination strategies to boost FcRn and Fc poly-reactivity of maternal IgG should continue to be considered. Further studies are needed to understand the mechanisms regulating selective placental antibody transport to develop strategies to modulate these factors for improved outcomes for pregnant women and their children.

## Materials and methods

### Study cohort samples

RV109 Plasma samples were collected from the RV109 MTCT cohort conducted in Lampang, Thailand between 1996-1998. The study enrolled drug naïve, CRF01_AE HIV-infected pregnant women and their infected or uninfected infants. Maternal samples used in the analysis were collected at delivery. Infant infection status was determined by DNA PCR using dried blood spot samples collected at 2, 4 and 6 months of life; infant samples used in the analysis were collected 2 months after birth. Maternal log_10_ viral loads ranged from 5.59 to 4.57 copies/ml, with a geometric mean of 5.07 copies/ml for T mothers, and 5.63 to 4.28 copies/ml, with a geometric mean of 4.73 copies/ml for NT mothers. Maternal CD4 counts ranged from 830 to 40 cells/mm^2^, with a geometric mean of 234 cells/mm^2^ for T mothers, and 960 to 100 cells/mm^2^, with a geometric mean of 396 cells/mm^2^ for NT mothers. The institutional review boards from Chiang Mai University and the Walter Reed Army Institute of Research (WRAIR) approved the study protocol.

### Production of mother and infant *env* IMC

RNA was extracted from mother or infant plasma utilizing the QIAamp Viral RNA Mini Kit (Qiagen, Hilden, Germany). Complementary DNA (cDNA) was synthesized from the RNA utilizing the Superscript III First-Strand Synthesis System for RT-PCR (Invitrogen, Waltham, MA), from which nested PCR was conducted using single genome amplification (SGA) strategy to retrieve the *env* gene. *Env* SGA amplicons were purified and sequenced by an ABI 3100 sequencer (Applied Biosystems, Waltham, MA). Sequences were analyzed using Sequencher software (Gene Codes Corporation, Ann Arbor, MI) (36, 37). SGA *env* from the same subject was pooled and then cloned into the pcDNA3.1/V5-His TOPO TA expression vector as previously described (38, 39). The vectors with SGA inserts were transformed into STBL2 cells (Thermo Fisher, Waltham, MA) and grown on ampicillin plates for 24 hours at 30°C. Individual bacterial colonies were individually picked and screened utilizing RT-PCR for *env* insert orientation with the SYBR GREEN kit (Thermo Fisher, Waltham, MA). Positive colonies were selected for miniprep purification using the PureYield Plasmid Miniprep System (Promega, Madison, WI) and re-sequenced to verify clone identity and lack of mutations. *Env* plasmids were co-transfected with pSG3ΔEnv (NIH HIV Reagent Program, Manassas, VA) into HEK239T cells to produce pseudovirus and infectivity was determined by TZM-bl cell infection. Chimeric IMC containing the Renilla luciferase reporter gene were produced incorporating one infectious *env* each from two mother, M036 and M071, and two infant, I036 and I038, into the 40061 major IMC genomic backbone as described (39, 40). Chimeric IMC clones were transfected in HEK293T cells and viral supernatants were screened for infectivity on TZM-bl cells. CM235 and TH023 IMC were previously produced.

### ADCC-Luc assay

ADCC was measured using the ADCC-Luc assay as previously described (41). The CEM.NKR CCR5+Luc+ cells (NIH HIV Reagent Program, Manassas, VA; target cells) were cultured in R10 medium and passaged 4 days before plating for infections. On the day of infection, 2 million cells with >95% viability were resuspended with 8mL of IMC diluted in R10 medium and 5µg/mL of DEAE-Dextran. Cells were plated in 6 well culture plates and incubated for 48 hours. The 6 IMCs used in this study included the Renilla luciferase reporter gene and each HIV env: TH023, CM235, M036, M071, I036 and I038. All IMC were determined to infect using the CCR5 receptor. Infected target cells were counted, viability was assessed, and infectivity was confirmed by Renilla luciferase detection. Required infectivity of each target cell treatment was 3-fold above background of uninfected cells and >75,000 relative light units (RLUs). Infected target cells were mixed with PBMC, that were rested overnight in R10 medium with 100 ng/mL IL-15 (Miltenyi Biotec, Bergisch Gladbach, Germany), at a ratio of 1:30. PBMC were previously obtained from a downselected HIV-negative donor by leukophoresis through the RV229b protocol approved by the WRAIR Institutional Review Board. In a half-area, white 96-well plate (VWR International, Radnor, PA), 25ul of cells, including 5,000 infected target cells and 150,000 PBMC, were mixed with 25ul of diluted mother (1:2025) or infant (1:450) plasma in duplicate. A positive control, HIVIG (NIH HIV Reagent Program, Manassas, VA), and negative control, IVIG (Sigma-Aldrich, St. Louis, MO), were utilized at a single concentration (25 µg/mL) in replicate. Plates were rotated at 300 rpm for 30 minutes at room temperature. Plates were centrifuged at 300 g for 1 minute and and then incubated at 37°C in 5% CO_2_ for 5 ½ hours. ViviRen live cell substrate (Promega, Madison, WI) was added to each well (50ul) and luciferase was detected using an Envision luminometer (Perkin Elmer, Waltham, MA). The negative cut-off value for the assay was 10% killing.

### ADCP assay

ADCP was measured using monocytic THP-1 cells (ATCC, Manassas, VA), as previously described (42). Briefly, CRF01_AE gp140 protein was biotinylated at a biotin to gp140 ratio of 50 following manufacturer’s instructions (Thermo Fisher, Waltham, MA) and excess biotin was removed using Zeba desalting columns (Thermo Fisher, Waltham, MA). Biotinylated gp140 was incubated with yellow-green streptavidin-fluorescent beads (Molecular Probes, Eugene, OR) for 2 hours at 37°C. The gp140 beads were diluted 100-fold. In a 96-well plate, 10μl of diluted beads were mixed with diluted mother (1:3000) or infant (1:1000) plasma then incubated 2h at 37°C. Healthy donor plasma was utilized as a negative control. THP-1 cells (20,000 cells per well) were added and plates were incubated at 37°C for 18 hours. Cells were fixed with 4% paraformaldehyde solution and fluorescence was evaluated on an LSRII flow cytometer (BD Bioscience, Franklin Lakes, NJ). The phagocytic score was calculated by multiplying the percentage of bead-positive cells by the geometric mean fluorescence intensity of the bead-positive cells and dividing by 10^4^.

### Total IgG quantitation

IgG subclass (IgG1-4) quantitation was conducted utilizing the Human IgG subclass profile kit (Thermo Fisher, Waltham, MA) following manufacturer’s instructions. Briefly, diluted concentration standards and plasma samples were added to wells coated with anti-IgG1, anti-IgG2, anti-IgG3, or anti-IgG4 and incubated for 30 minutes. Wells were then washed and 100 µL of peroxidase anti-human IgG was added to each well and incubated for 30 minutes. After washing, 100 µL of 5,5′-Tetramethylbenzidine (TMB) solution was added and incubated for 10 minutes. The reaction was stopped by adding 100 µL of stop solution. Absorbance of each well was measured at 450 nm using a VersaMax (Molecular Devices, San Jose, CA) and IgG subclass concentrations were calculated using a standard curve.

### Gp140 protein production

For this study, gp140 proteins were produced for two mothers, M036 and M071, and two infants, I036 and I038. Other gp140 proteins utilized in this study were produced using the same method, as described previously (43). Briefly, uncleaved gp140 proteins were designed with R to S mutations in the gp120/gp41 cleavage site, incorporated native leader peptide and full MPER sequence followed by a short GGGS linker sequence, and a C-terminal AviTag. Sequences were codon-optimized for expression in human cells, synthesized (Genscript, Piscataway, NJ), and cloned into a custom pcDNA3.4 (Thermo Fisher, Waltham, MA) expression vector. Proteins were expressed by transient transfections in Expi293 cells (Thermo Fisher, Waltham, MA) according to the manufacturer’s instructions. Gp140 proteins were purified from clarified cell culture supernatants 4 days post-transfection using Galanthus nivalis lectin (GNL) affinity chromatography (Vector Laboratories, Burlingame, CA). Proteins were further flowed through a Q-sepharose (GE Healthcare, Chicago, IL) column to remove host cell protein contaminants. All gp140s were purified to 90% purity or higher, as assessed by SDS-PAGE and Coomassie staining in reducing and non-reducing conditions.

### Luminex multiplex assay

Antibody binding and Fc characterization was determined by Luminex multiplex assay, as previously described (44–46) with minor modifications. Heat-inactivated plasma was diluted to 1:100 and 1:100 and was loaded in duplicate into 384-well assay plates by a Biomek NXP automated liquid handler (Beckman Coulter, Brea, CA). HIV infected and uninfected plasma were used as positive and negative controls, respectively. Antigen-coated microspheres (Luminex Corporation, Austin TX) were added and plates were incubated for 2 hours with vigorous shaking. Microspheres were washed then resuspended with 40 μL R-phycoerthrin (PE)-labeled Fc detection reagent for 1 hour. Microspheres were washed then resuspended in 40 μL sheath fluid (Luminex Corporation, Austin TX) and analyzed on a Bio-Plex 3D Suspension Array system (Bio-Rad, Hercules CA) running xPONENT v.4.2 (Luminex Corporation, Austin, TX).

Antigen-coated microspheres: Antigens evaluated in this study included two gp120 proteins (strains TH023 and A244), six gp140 proteins (strains 40512, 40646, I036, I038, M036, M071), two gp70-scaffolded V1V2 proteins (strains TH023 and A244), two gp70-scaffolded V3 proteins (strains A244 and A244/TH023) and one gp41 protein (strain MN). All antigens were subtype CRF01_AE, with the exception of subtype B gp41 MN. Proteins were produced for this study, or obtained from Southern Biotech (Birmingham, AB), the Duke University Protein Production Facility (Durham, NC) or the NIH AIDS Reagent Program (Bethesda, MD). Antigens were covalently coupled to uniquely coded carboxylate magnetic microspheres (Luminex Corporation, Austin TX), as described previously (46). Microspheres were incubated for 20 minutes in buffer containing 1-Ethyl-3 [3-dimethylaminopropyl]carbodiimide hydrochloride and N-hydroxysulfosuccinimide. Each protein antigen was added to a uniquely coded microsphere and incubated for 2 hours. Beads were washed and stored at -80°C in PBS containing 0.1% BSA, 0.05% sodium azide and 0.02% Tween-20. Coupling efficiency and specificity were confirmed by testing against a panel of HIV infected and healthy control plasmas.

IgG subclass and Fc detectors: Ig subclass was determined by detection with PE-conjugated mouse anti-human IgG, and IgG1-4 (Southern Biotech, Birmingham, AL). Expression vectors for human Fc receptors, including, each constructed with a C-terminal AviTag, were produced *via* transient transfection and purification over Ni-NTA (Qiagen, Germantown, MD) affinity column and gel filtration on an Enrich SEC70 column (Bio-Rad, Hercules, CA). Fc receptor, including FcRn, FcγRIIA(H131), FcγRIIA(R131), FcγRIIB, FcγRIIIA(F158), FcγRIIIA(V158), FcγRIIIB(NA1), FcγRIIIB(NA2), and FcγRIIIB(SH) were biotinylated using the BirA Biotin-protein ligase reaction kit (Avidity, Aurora, CO).

### PBMC Genotyping analysis

Custom TaqMan SNP Genotyping Assays (ThermoFisher Scientific, Waltham, MA) were performed per manufacturer’s recommendations to differentiate SNP variants in FcγR2A (Assay ID: C_9077561_20) or FcγR3A (Assay ID: C_25815666_10) genes at positions rs1801274 and rs396991, respectively. Briefly, duplicate reactions were set-up in MicroAmp Optical 384-well plates (ThermoFisher, Waltham, MA) in 5ul volumes consisting of 10 ng gDNA, 2.5 ul 2X Universal PCR Master Mix-No AmpErase UNG (ThermoFisher Scientific, Waltham, MA), 0.0625 ul relevant 40X TaqMan SNP Assay Mix, and 1.4375 ul water. PCR was performed using the following parameters on the 7900HT Fast Real-Time PCR System and SDS2.4 software (ThermoFisher Scientific, Waltham, MA) with the Standard Curve (AQ) Assay and Standard Mode Protocol settings: 95°C, 10 minutes; 40 cycles of 92°C, 15 seconds; 60°C, 60 seconds. Post-PCR, plates were read and analyses were performed through the Allelic Discrimination Assay set-up.

### PBMC receptor expression characterization

Cryopreserved infant PBMC cells were thawed, washed, counted and viabilities were obtained using Guava Viacount reagent (Luminex Corporation, Austin, TX). PBMC were washed with 10 mL of PBS before staining with Aqua Live/Dead (ThermoFisher Scientific, Waltham, MA) for 30 minutes at room temperature, in the dark. Cells were washed then blocked with 10% normal mouse IgG diluted in staining buffer and incubated at room temperature for 15 minutes. Cells were washed again and surface stained with a cocktail of fluorochrome conjugated antibodies for 30 minutes at room temperature in the dark. Cells were washed, fixed with 2% PFA, and incubated at room temperature for 15 minutes. Cells were washed and read on an LSRII flow cytometer (BD Bioscience, Franklin Lakes, NJ). Data was analyzed using FlowJo v10 software.

### Statistical methods

Prism v.8.0.2 (GraphPad Software, Inc., San Diego, CA) and R(3.6.3) through R Studio (1.1.453, R Consortium, Boston, MA) were employed for statistical analyses and graphical representations of data. Data outliers identified in the binding antibody analyses were excluded by local outlier factor method. Univariate binding, ADCC and ADCP analyses were assessed for statistical differences by Mann-Whitney (two-tailed) test. Binding correlations were conducted by Spearman’s rank-order correlation (two-tailed). An unsupervised principle component analysis was performed to determine which antibody profiling feature best contributed to explaining the variance between mother and infant groups. Each dot in the plot represents a mother or infant in study. Ellipses correspond to 95% confidence intervals for each group. Variable Important Projection (VIP) scores were generated through Partial Least Square Discriminant Analysis (PLSDA). PLSDA models were generated for predictive analysis to assess ability of features to classify the mother and infant groups. VIP scores indicate the capability of contributing to the model in terms of separating transmitting from non-transmitting mothers and infected from uninfected infants. VIP score > 1 was used as threshold to select the most contributing features among all binding features in study. Area under the receiver operating characteristic curve (AUROC) was generated to predict the ability of selected features to separate mothers by transmission status and infants by infection status.

## Data availability statement

The raw data supporting the conclusions of this article will be made available by the authors, without undue reservation.

## Ethics statement

The institutional review boards from Chiang Mai University and the Walter Reed Army Institute of Research (WRAIR) approved the study protocol. Written informed consent to participate in this study was provided by the participants’ legal guardian/next of kin.

## Author contributions

MR, ST, and KS oversaw the RV109 clinical trial. BB, SK, ST, VP, and LW designed the study. BB, DC, AO’S, BM, ES-B, AL-C, and ST planned and produced IMC constructs. SK, BS, VD, UT, and LM-R performed protein production and Luminex analysis. BB and MZ performed ADCC experiments. DP-P and ZS performed ACDP experiments. PE and RT performed genetic analysis. ST, VP, LW, SK, VR, ES-B, DP-P, GF, RT, MC, ME, NJ, and AL-C provided expert guidance. BB, NJ, and LW performed statistical analysis. BB, VP, MZ, and LW wrote the manuscript. All authors contributed to the article and approved the submitted version.

## Supplementary material

Supplementary Figure 1 Mother and infant plasma ADCP activity. ADCP activity was measured for the **A**) transmitting (T) and non-transmitting (NT) mothers and **B**) HIV+ and uninfected (HIV-) infants using a cohort maternal HIV M036 strains gp140 protein. Specific signal to background noise (S:N) ratios are reported.

Supplementary Figure 2 Paired mother and infant binding antibody responses. Plasma antibody responses were compared between paired mothers and infants. Total IgG and FcRn-specific IgG were evaluated using the geometric mean of the binding antibody mean florescence intensity (MFI) to six gp140 proteins, two V1V2 proteins, and two V3 antigens. Total IgG responses are shown for A) gp140, B) V1V2 and C) V3. FcRn-specific IgG responses are shown for D) gp140, E) V1V2 and F) V3. Transmitting (T) pairs, consisting of a T mother and her HIV+ infant, are shown in red; nontransmitting (NT) pairs, consisting of a NT mother and her HIV- infant, are shown in gray.

Supplementary Figure 3 Infant PBMC genotyping and receptor expression. HIV+ and uninfected (HIV-) infant PBMC were sequenced to determine the allotypic variants in the FcγR3a receptors at position 158 (V or F). Infant PBMC were immunophenotyped for NK maturation markers that are indicative of C) cytoxic (CD56dim) and D) highly cytotoxic (CD56dim+/CD57+) populations.
