# Supplementary figures and images for: Fc receptor engagement of HIV-1 Env-specific antibodies in mothers and infants predicts reduced vertical transmission
Source: Front Immunol. 2022 Dec 12;13:1051501. doi: 10.3389/fimmu.2022.1051501 (PMC9791209; doi:10.3389/fimmu.2022.1051501)

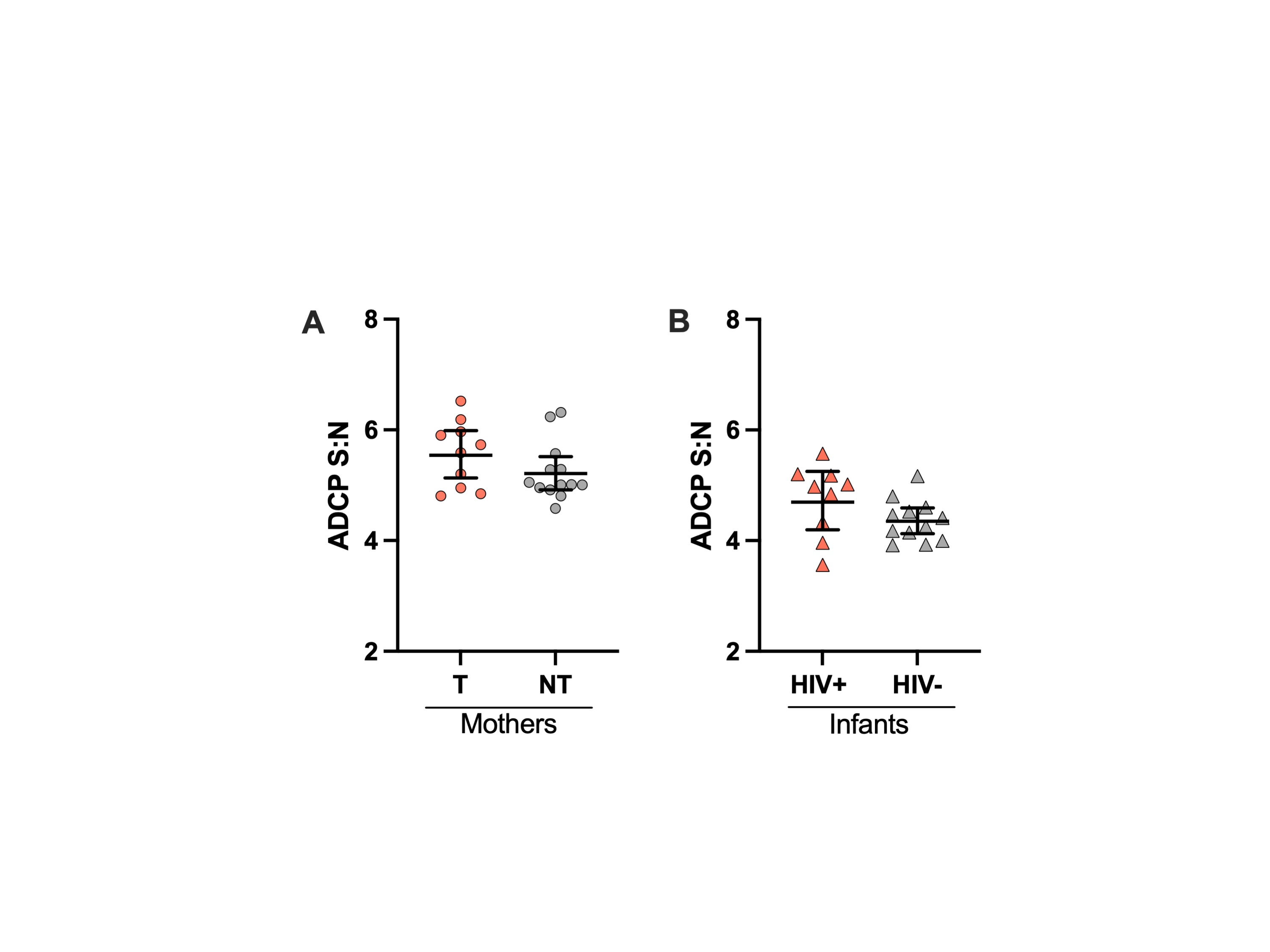

Supplement: Supplementary Figure 1 — Mother and infant plasma ADCP activity. ADCP activity was measured for the A) transmitting (T) and non-transmitting (NT) mothers and B) HIV+ and uninfected (HIV-) infants using a cohort maternal HIV M036 strains gp140 protein. Specific signal to background noise (S:N) ratios are reported. [file Image_1.jpeg]

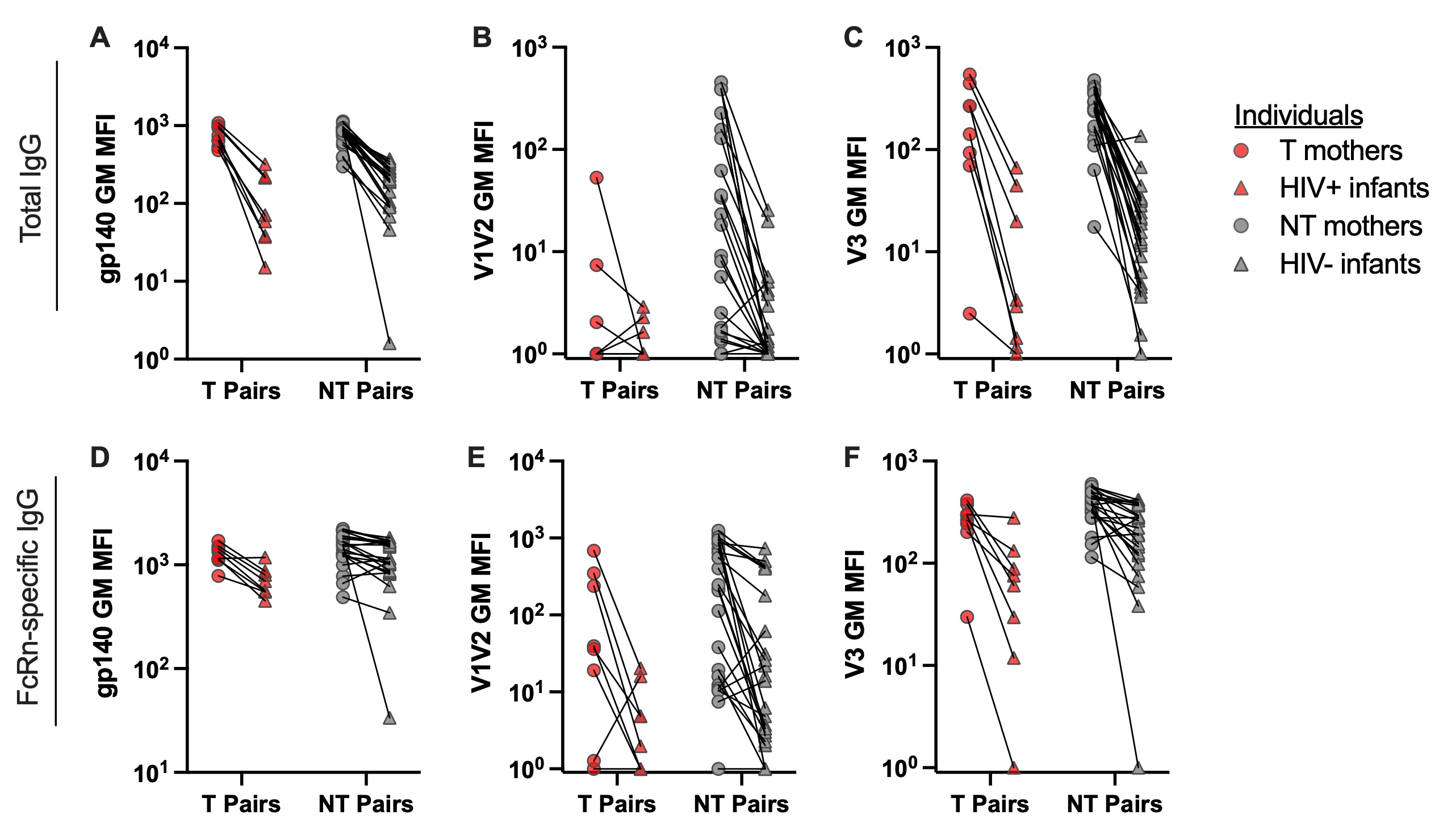

Supplement: Supplementary Figure 2 — Paired mother and infant binding antibody responses. Plasma antibody responses were compared between paired mothers and infants. Total IgG and FcRn-specific IgG were evaluated using the geometric mean of the binding antibody mean florescence intensity (MFI) to six gp140 proteins, two V1V2 proteins, and two V3 antigens. Total IgG responses are shown for A) gp140, B) V1V2 and C) V3. FcRn-specific IgG responses are shown for D) gp140, E) V1V2 and F) V3. Transmitting (T) pairs, consisting of a T mother and her HIV+ infant, are shown in red; nontransmitting (NT) pairs, consisting of a NT mother and her HIV- infant, are shown in gray. [file Image_2.jpeg]

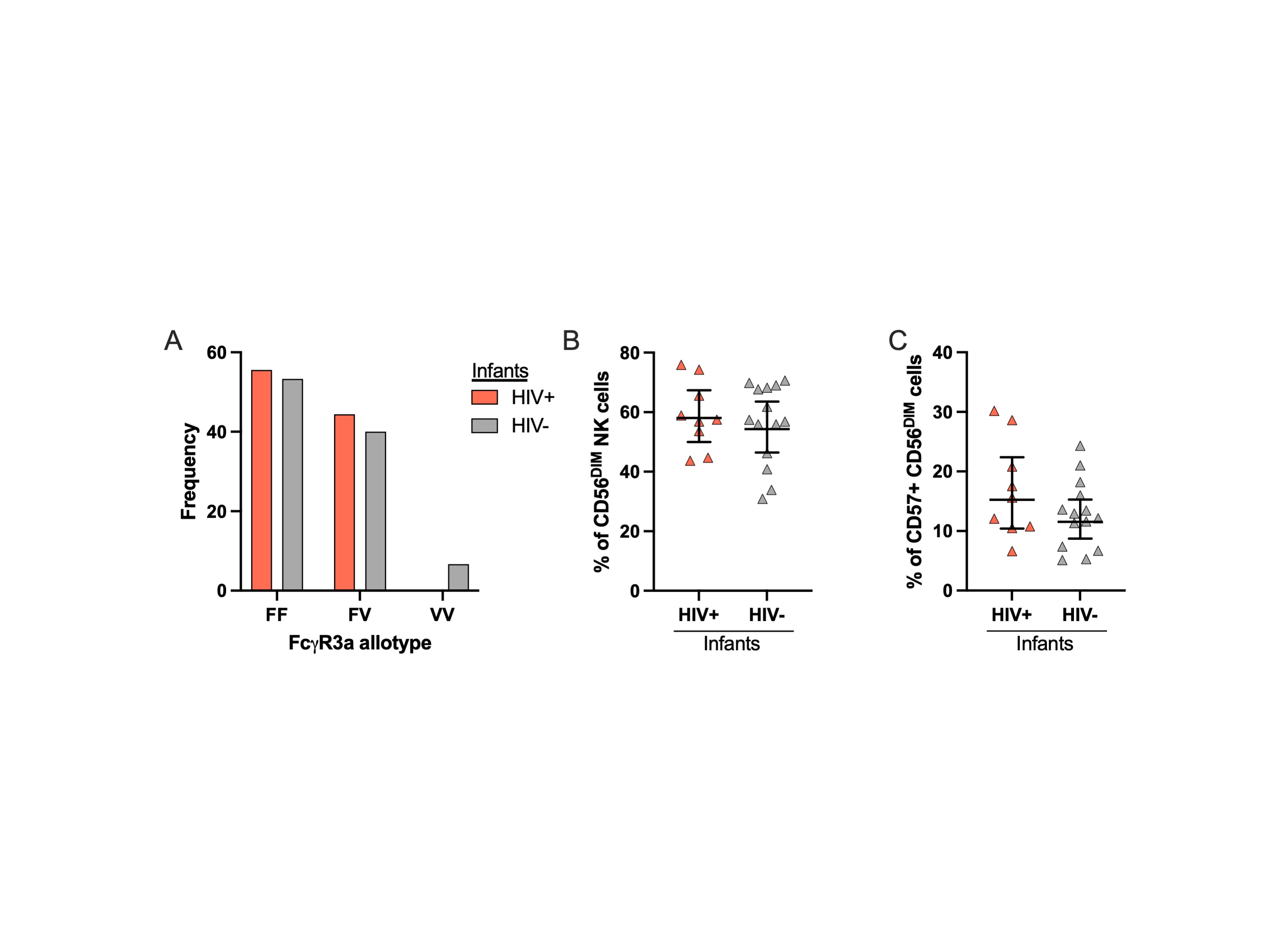

Supplement: Supplementary Figure 3 — Infant PBMC genotyping and receptor expression. HIV+ and uninfected (HIV-) infant PBMC were sequenced to determine the allotypic variants in the FcγR3a receptors at position 158 (V or F). Infant PBMC were immunophenotyped for NK maturation markers that are indicative of C) cytoxic (CD56dim) and D) highly cytotoxic (CD56dim+/CD57+) populations. [file Image_3.jpeg]
